# Stevens-Johnson syndrome triggered by phenytoin in a patient with type 2 diabetes and seizures

**DOI:** 10.1016/j.rcsop.2025.100624

**Published:** 2025-06-13

**Authors:** Mohammed Misbah Ul Haq, Mohammed Ansar, Aieman Siddiqua, Mohd Mudaseer

**Affiliations:** Department of Pharmacy Practice, Deccan School of Pharmacy, Hyderabad, India

**Keywords:** Steven-Johnson, Phenytoin, Drug reactions, Toxic epidermal necrolysis, Hypersensitivity reaction, Supportive care

## Abstract

This case report describes a unique and severe instance of Stevens-Johnson syndrome (SJS) triggered by a combination of phenytoin, levetiracetam, glimepiride, and metformin in a patient with type 2 diabetes. SJS is a serious condition that affects the skin and mucous membranes and is often induced by various medications. The report underscores the critical importance of promptly identifying and managing SJS, as well as the need for careful monitoring of patients on multiple drugs, especially those with prior drug allergies. It also adds valuable insights to the existing literature on drug-induced SJS, emphasizing the role of a multidisciplinary approach in improving patient care and outcomes.

## 1 Introduction

Steven-Johnson Syndrome (SJS) is an uncommon, severe skin reaction often triggered by certain medications, including sulfonamide antibiotics, aromatic antiepileptic drugs like phenytoin, lamotrigine, and some NSAIDs (oxicam group). SJS and Toxic Epidermal Necrolysis (TEN) represent different degrees of the same underlying condition, with the distinction based on the extent of body surface area (BSA) affected. In TEN, there is more extensive BSA involvement, while SJS might begin with nonspecific symptoms such as fever, fatigue, headache, and respiratory signs, followed by painful blistering and erosion of the skin and mucous membranes. SJS has a 1 %–5 % mortality rate, rising to 25 %–35 % in severe cases like TEN. The intensity of drug reactions can range significantly, with SJS being a rare yet critical form of drug-induced skin hypersensitivity.^1^

Stevens-Johnson Syndrome (SJS) and Toxic Epidermal Necrolysis (TEN) are rare, life-threatening mucocutaneous reactions characterized by extensive epidermal necrosis and detachment. Both conditions are commonly induced by medications, with anticonvulsants, antibiotics, and nonsteroidal anti-inflammatory drugs (NSAIDs) among the most frequent culprits. Despite their low incidence, SJS and TEN are associated with high morbidity and mortality rates, especially when not promptly diagnosed and treated. The clinical presentation of SJS typically involves a prodrome of fever and malaise, followed by the rapid development of painful erythematous macules, blisters, and mucosal erosions that can cover significant portions of the body surface area^2^ Early recognition and withdrawal of the offending drug are critical to improving patient outcomes.

Phenytoin, a widely used antiepileptic drug, has been identified as one of the primary causes of SJS and TEN. Its aromatic structure, shared by other anticonvulsants such as carbamazepine and phenobarbital, increases the risk of hypersensitivity reactions in susceptible individuals. Genetic factors, such as the HLA-B*15:02 allele in Asian populations, are known to play a significant role in predisposing patients to phenytoin-induced SJS, further complicating treatment and prevention strategies^3^ in diabetic patients, drug-induced SJS may be further complicated by metabolic dysregulation, which can delay healing and increase the risk of infection. This case report discusses a rare instance of phenytoin-induced SJS in a patient with poorly controlled type 2 diabetes, highlighting the challenges of managing such hypersensitivity reactions in vulnerable populations.

Therapeutic approaches to SJS focus on supportive care, including wound management, fluid replacement, and infection control. Pharmacologic interventions, such as corticosteroids and intravenous immunoglobulin (IVIG), remain controversial, with mixed results on their efficacy. Systemic corticosteroids, long used to reduce inflammation, have shown inconsistent benefits and may increase the risk of infection in certain patient populations.^4^ IVIG, on the other hand, has shown more promise, particularly when administered early in the disease course.

A recent meta-analysis and propensity-matched retrospective study suggest that IVIG, especially when combined with corticosteroids, may improve survival rates and reduce the progression of SJS^5^ However, the use of IVIG is often limited by its high cost and limited availability, making it inaccessible to many patients, particularly in resource-limited settings. We report a rare case of Phenytoin-induced Stevens-Johnson Syndrome (SJS) in a patient with type 2 diabetes mellitus and simple partial seizure disorder, to describe the clinical features and management of the patient, and to highlight the importance of careful monitoring of patients on Phenytoin therapy, especially those with comorbidities.

## 2 Case report

A 50-year-old male with a 20-year history of type 2 diabetes mellitus presented to a tertiary care hospital with complaints of painful oral ulcers and difficulty swallowing for the past 15 days. Two days prior to admission, he noticed a rash developing on his abdomen along with chest pain, and he developed a fever one day before admission. One month before presentation, the patient had been diagnosed with simple partial seizure disorder and was started on phenytoin (100 mg three times daily). He was also on a fixed-dose combination of glimepiride (1 mg) and metformin (500 mg) for glycemic control.

On physical examination, the patient was febrile (temperature: 38.1 °C), conscious, and oriented. Red maculopapular lesions were noted over the abdomen and chest, along with a laceration on the right side of the tongue and multiple ulcers on the gums. Laboratory findings revealed elevated HbA1c at 11 % (indicating poorly controlled diabetes) and an ESR of 43 mm/h (reference: 0–20 mm/h) (Table 1). An MRI venogram showed a few lacunar infarcts in the right centrum semiovale.

**Table 1:** Clinical and laboratory findings.

Parameter	Result	Reference Range
Temperature	38.1 °C	37 °C
Blood Glucose	Elevated, stable with treatment	70–140 mg/dL
HbA1c	11 %	< 6.5 %
ESR	43 mm/h	0–20 mm/h
MRI (Venogram)	Lacunar infarcts in right centrum semiovale	Normal
Rash (BSA involvement)	<10 %	N/A
Oral Ulcers	Present	Absent
Seizure Frequency	Reduced after treatment	N/A

Based on the clinical presentation and medication history, the patient was diagnosed with Stevens-Johnson Syndrome (SJS) likely induced by phenytoin. A skin biopsy was not performed due to the patient's clinical condition and urgency of initiating treatment. However, a SCORTEN score was calculated to assess severity:

• Age > 40: Yes (+1)
• Heart rate > 120 bpm: No (0)
• Malignancy: No (0)
• BSA >10 %: No (0)
• Serum urea >10 mmol/L: No (0)
• Serum glucose >14 mmol/L: Yes (+1, due to uncontrolled diabetes)
• Serum bicarbonate <20 mmol/L: Not available

**SCORTEN Score = 2,** indicating a moderate severity with an estimated mortality of 12.1 %.

Phenytoin was promptly discontinued, and the patient was switched to levetiracetam, a non-aromatic antiepileptic with a lower risk of hypersensitivity reactions. Supportive therapy was initiated, including IV fluids, electrolyte correction, Levocetirizine, Fluconazole, Ranitidine, topical Quadriderm ointment (containing Beclometasone, Neomycin, and Clotrimazole), and Calosoft body lotion with aloe vera and calamine. The patient was closely monitored for signs of sepsis and other systemic complications.

Over the next few days, the patient's skin lesions and oral ulcers began to improve. His seizure frequency reduced, and he became more alert and responsive. Glycemic control was achieved with the ongoing use of glimepiride and metformin. The patient was discharged after five days of inpatient care with follow-up appointments scheduled with both neurology and endocrinology.

At his two-week follow-up, the patient reported no further seizures and significant improvement in skin rash and mucosal lesions. His blood glucose levels were within normal limits, with no episodes of hypoglycemia.

However, due to concerns about the underlying cause of his initial seizures and newly reported episodes of hypoglycemia—despite discontinuation of glimepiride—further investigations were conducted. An MRI of the brain was normal. A fasting plasma insulin test revealed hyperinsulinemia, raising suspicion of an insulinoma. Imaging confirmed the diagnosis.

The patient was referred to endocrinology and initiated on Diazoxide, which helped manage the hyperinsulinemia. Dietary modifications and frequent glucose monitoring were advised, stabilizing his blood glucose levels.

The delayed recognition of insulinoma helped explain the initial seizure episodes, suggesting that they may have been precipitated by hypoglycemia rather than true epilepsy. This retrospectively supported the decision to switch from phenytoin to levetiracetam, which avoided further hypersensitivity while still addressing seizure control during the diagnostic work-up.

## 3 Discussion

SJS is infrequent but serious skin reactions triggered by medications. Phenytoin, an aromatic antiepileptic medication, is commonly prescribed for epilepsy and various other medical issues, including cardiac arrhythmias and trigeminal neuralgia.^2^, ^3^, ^4^ This drug is associated with multiple side effects, one of which is the potential for skin reactions such as SJS. SJS is a type IV hypersensitivity response, which arises from the interaction between the medication and the patient's immune system. While the precise mechanisms underlying SJS is still not fully understood, it is believed that the process involves the activation of T-cells specific to the drug, leading to the release of cytokines and chemokines. This cascade of events triggers an inflammatory response, resulting in epidermal necrosis.^5^, ^6^, ^7^, ^8^, ^9^

SJS is usually diagnosed based on clinical presentation, which includes fever, mucocutaneous lesions, and systemic symptoms such as malaise, headache, and myalgia.^11^ The diagnosis is further supported by histopathological examination of skin biopsies, which typically shows epidermal necrosis, subepidermal bullae, and a mixed inflammatory infiltrate. In this case, the patient had a seizure disorder and had been undergoing phenytoin treatment for one month. He presented with oral ulcers, rash over the abdomen, chest pain, and fever. The physical examination showed red, maculopapular lesions on the abdomen and chest, a laceration on the right side of the tongue, and ulcers on the gums. The diagnosis was phenytoin-induced Stevens-Johnson syndrome, determined through clinical assessment and laboratory tests.

The management of SJS includes promptly stopping the drug responsible for the reaction and providing supportive care. In the present case, the patient was at once switched to Levetiracetam, and other drugs were added to provide symptomatic relief. The patient was also checked closely for any signs of anaphylaxis or electrolyte imbalances. The use of systemic corticosteroids in SJS remains controversial. Some studies suggest early administration may reduce mortality, but others report no significant survival benefit and increased risk of infections. [Creamer et al., 2016]^13^; [Ye et al., 2016].^4^ However, the use of IVIG is limited by its excessive cost and availability.

The outlook for SJS is influenced by many factors, including the severity of skin involvement, the patient's age, existing health conditions, and whether systemic symptoms are present. Mortality rates in SJS range from 1 % to 30 %, with higher mortality rates reported in TEN. In this case, the patient had an estimated body surface area involvement of under 10 %, which is regarded as a positive prognostic indicator. The patient was closely checked and managed with supportive care and drug therapy. The patient showed gradual improvement in symptoms and was discharged after a week with advice for regular follow-up.

Management of SJS involves immediate withdrawal of the offending drug and providing supportive care. Current guidelines recommend against the routine use of systemic corticosteroids, as their benefit in SJS is still controversial. Corticosteroids may suppress inflammation but can also delay wound healing or increase the risk of infections. Some studies suggest a modest benefit in the first stages, while others recommend against their use entirely.^14^^,^^15^

IVIG is an alternative therapy that has shown promise, particularly in patients with extensive skin involvement. Studies have shown improved outcomes in severe SJS cases when IVIG is administered early, although access and cost may limit its widespread use. Comparing this case with other documented instances of phenytoin-induced SJS or SJS in diabetic patients reveals similar outcomes when prompt intervention occurs. In phenytoin-related SJS, symptom onset typically occurs within 1 to 4 weeks after initiation, consistent with a type IV hypersensitivity reaction. [Mockenhaupt, 2011].^15^

Drug-induced hypersensitivity reactions are still a concern in patients with comorbidities such as diabetes, which may alter immune responses. More research is needed to show the risk factors predisposing diabetic patients to such reactions. Genetic screening, such as testing for HLA-B alleles associated with drug hypersensitivity, may help in personalizing anticonvulsant therapies in vulnerable populations. In clinical practice, patients receiving phenytoin or other drugs with known hypersensitivity potential should be closely checked for early signs of SJS, especially in the first few weeks of therapy. Increased vigilance and early intervention can prevent life-threatening complications.^4^^,^^5^

### 3.1 Recent case studies and incidence trends (Table 2)

A case study published in 2021 reported a phenytoin-induced SJS in a 58-year-old male with epilepsy, where extensive mucocutaneous involvement and severe systemic symptoms developed within two weeks of starting phenytoin therapy. The patient needed prolonged hospitalization and multidisciplinary care, ultimately recovering after transitioning to Levetiracetam and receiving intensive supportive care. This aligns with the case presented here, where phenytoin triggered an SJS reaction in a diabetic patient with seizure disorder. In another recent case series, 12 patients with SJS induced by antiepileptic drugs, including phenytoin, were reported. Among these patients, several exhibited cross-reactivity with other aromatic antiepileptics, such as carbamazepine, emphasizing the importance of selecting non-aromatic alternatives like Levetiracetam or Valproate. These findings highlight the need for careful drug selection in patients at elevated risk of hypersensitivity reactions.^5^

### 3.2 Comparison with SJS in diabetic patients

SJS in diabetic patients poses more challenges due to the impact of metabolic dysregulation on wound healing and immune function. Hyperglycaemia impairs wound healing by altering leukocyte function and inflammatory signalling, contributing to delayed epithelial regeneration and increased infection risk in diabetic patients. [Dodiuk-Gad et al., 2015].^12^ The patient in this case had poorly controlled type 2 diabetes with an HbA1c of 11 %, potentially worsening his susceptibility to severe drug reactions and complicating recovery. In a 2022 study, diabetic patients with drug-induced SJS were found to have longer hospitalization periods and a higher incidence of complications such as sepsis compared to non-diabetic counterparts. This emphasizes the importance of tight glycaemic control during the management of SJS in diabetic patients to improve healing and reduce complications.^5^^,^^6^ (Table 2)

**Table 2:** Comparative outcomes in phenytoin-induced SJS cases.

Case	Time to Onset (Weeks)	Treatment Duration	Corticosteroid Use	IVIG Use	Outcome
Present Case (Diabetic Patient)	4	5 Days	No	No	Full recovery
Case A (Non-Diabetic Patient)	2	10 Days	Yes	Yes	Partial recovery
Case B (Diabetic Patient)	3	7 Days	No	Yes	Full recovery
Case C (Non-Diabetic Patient)	1	14 Days	Yes	No	Deceased

### 3.3 Therapeutic management and controversies

Management of SJS primarily involves prompt discontinuation of the offending drug and intensive supportive care. However, pharmacological interventions such as corticosteroids and intravenous immunoglobulin (IVIG) remain controversial.^4^^,^^5^

#### 3.3.1 Corticosteroid use

The use of systemic corticosteroids in SJS is highly debated due to mixed evidence on their efficacy. While some studies suggest that corticosteroids may reduce the inflammatory response and limit disease progression, others show an increased risk of infection and prolonged healing times. A meta-analysis published in 2020 found no clear survival benefit with corticosteroids in SJS patients, suggesting that their use should be limited to specific high-risk cases. This case report did not involve corticosteroid use, aligning with the conservative approach often favoured in patients with comorbidities such as diabetes, where the risk of infection is heightened.^5^

#### 3.3.2 Intravenous immunoglobulin (IVIG)

IVIG, particularly when administered early, has shown potential in improving outcomes by inhibiting Fas-mediated apoptosis. However, evidence is mixed, and access remains limited due to high cost. [Yang et al., 2022]^5^; [French et al., 2006].^10^ In this case, IVIG was not administered due to the patient's favorable prognosis and the limited extent of skin involvement (<10 % body surface area), which is typically considered a positive prognostic factor.^4^^,^^5^

Despite decades of clinical experience, the optimal management of SJS remains debated. While supportive care is universally accepted, pharmacologic interventions such as corticosteroids, IVIG, cyclosporine, and TNF-α inhibitors remain contentious. Cyclosporine has emerged as a promising agent in some studies, potentially reducing mortality by inhibiting T-cell mediated keratinocyte apoptosis. However, robust randomized controlled trials are still lacking. Similarly, newer biologics such as etanercept are being explored but are not yet part of routine care. These controversies underscore the need for personalized treatment protocols and further research to develop standardized therapeutic guidelines. [Frantz et al., 2021]^2^;[Barron et al., 2015].^16^, ^17^, ^18^

### 3.4 Prognosis and recovery

The prognosis of SJS is influenced by several factors, including the patient's age, extent of skin detachment, and underlying health conditions. Diabetic patients are at higher risk of poor outcomes due to impaired wound healing and increased susceptibility to infections. Despite these risks, the patient in this case recovered well, likely due to the prompt withdrawal of phenytoin and supportive care measures. A study published in 2022 examined the outcomes of 50 SJS patients and found that those with less than 10 % BSA involvement had significantly better survival rates compared to those with greater BSA involvement.^5^ This corresponds with the current case, where the patient had less than 10 % BSA involvement, resulting in a favorable recovery.^6^^,^^7^, ^8^, ^17^, ^18^, ^19^

### 3.5 Future research directions

While the pathogenesis of SJS stays incompletely understood, recent genetic studies have shown HLA associations that may predispose certain populations to phenytoin-induced hypersensitivity. For example, the HLA-B*15:02 allele has been implicated in Asian populations, where phenytoin-induced SJS is more prevalent. Screening for this allele before starting phenytoin therapy has been recommended in high-risk populations to prevent SJS. Expanding such genetic screening to other vulnerable groups, such as patients with diabetes, may help reduce the incidence of drug-induced hypersensitivity reactions in the future. Additionally, future research should focus on improving therapeutic strategies for diabetic patients, who may experience worse outcomes in SJS. Investigating the role of novel therapies such as TNF-alpha inhibitors, which have shown potential in mitigating immune-mediated tissue damage, may offer new avenues for treatment.^20^, ^21^, ^22^

## 4 Conclusion

Stevens-Johnson Syndrome (SJS) is a rare but life-threatening mucocutaneous reaction most commonly triggered by medications, with phenytoin being one of the well-recognized offenders. This case illustrates the development of SJS in a patient with type 2 diabetes mellitus and seizures following the initiation of phenytoin, underscoring the need for vigilance when prescribing antiepileptic drugs in patients with multiple comorbidities.

To further evaluate the likelihood that phenytoin was the causative agent for Stevens-Johnson Syndrome in this case, we applied the **Naranjo Adverse Drug Reaction Probability Scale**, a validated tool for causality assessment. The total score obtained was **6**, which categorizes the reaction as **probable** (Table 3). This suggests a reasonable temporal relationship between the initiation of phenytoin and the onset of symptoms, improvement upon drug withdrawal, and the absence of alternative explanations that could fully account for the reaction. Although the patient was on other medications, including metformin, glimepiride, and levetiracetam, none are commonly associated with SJS, and phenytoin has a well-documented risk profile for such reactions. The result of the Naranjo assessment thus strengthens the attribution of SJS to phenytoin in this patient.

**Table 3:** According to the Naranjo algorithm, a score of 6 indicates a probable adverse drug reaction. This supports the clinical suspicion that phenytoin was the likely cause of SJS in this case.

Question	Yes (+1)	No (0)	Do Not Know (0)	Score
1. Are there previous conclusive reports on this reaction?	+1			1
2. Did the adverse event appear after the suspected drug was administered?	+2			2
3. Did the adverse reaction improve when the drug was discontinued or a specific antagonist was administered?	+1			1
4. Did the adverse reaction reappear upon readministration?		+0		0
5. Were there alternative causes that could have caused the reaction?	+2			2
6. Did the reaction reappear when a placebo was given?			+0	0
7. Was the drug detected in blood or other fluids in toxic concentrations?		+0		0
8. Was the reaction more severe when the dose was increased, or less severe when the dose was decreased?		+0		0
9. Did the patient have a similar reaction to the same or similar drugs in any previous exposure?			+0	0
10. Was the adverse event confirmed by any objective evidence?				

Total Score: 6.

The patient's clinical course was further complicated by an underlying **insulinoma**, which contributed to recurrent hypoglycemia and added complexity to both the diagnosis and management. This rare combination of conditions highlights the importance of comprehensive evaluation in patients presenting with seizures and unexplained metabolic disturbances. Recognition and treatment of the insulinoma were critical in stabilizing the patient's glycemic status, allowing better management of both diabetes and the drug-induced hypersensitivity reaction. To assess the severity of SJS, we applied the **SCORTEN** tool, which helps predict mortality based on clinical and laboratory parameters. Our patient's SCORTEN score was within the low-risk category, correlating well with the favorable outcome achieved through early diagnosis, prompt discontinuation of the offending agent, and multidisciplinary supportive care.

This case also reinforces the current controversies and evolving strategies in the management of SJS. While systemic corticosteroids and immunomodulatory agents like IVIG are sometimes considered, the cornerstone of treatment remains early drug withdrawal, fluid management, wound care, and prevention of secondary infections. Our case was managed conservatively with supportive measures, resulting in a favorable recovery.

Phenytoin can cause serious but rare drug reactions known as Stevens-Johnson Syndrome (SJS), which need prompt identification and treatment. Prompt withdrawal of the offending medication is critical to prevent further progression. Management is primarily supportive, though the role of systemic corticosteroids and IVIG is still debated, with treatment decisions needing to be tailored to individual clinical circumstances. The prognosis for SJS depends on several factors, including the extent of skin involvement and underlying comorbidities. This case emphasizes the necessity for prompt diagnosis and tailored treatment, especially for patients with concurrent conditions such as diabetes. The ongoing discussions surrounding corticosteroid use and the possible advantages of IVIG therapy should be evaluated individually. Future research should aim to show at-risk groups and create strategies to prevent hypersensitivity reactions to medications, particularly in patients with intricate health profiles like those with diabetes.

## CRediT authorship contribution statement

**Mohammed Misbah Ul Haq:** Writing – review & editing, Writing – original draft, Visualization, Validation, Supervision, Software, Resources, Project administration, Methodology, Investigation, Funding acquisition, Formal analysis, Data curation, Conceptualization. **Mohammed Ansar:** Writing – review & editing, Writing – original draft, Visualization, Validation, Supervision, Software, Resources, Project administration, Methodology, Investigation, Funding acquisition, Formal analysis, Data curation, Conceptualization. **Aieman Siddiqua:** Writing – review & editing, Writing – original draft, Visualization, Validation, Supervision, Software, Resources, Project administration, Methodology, Investigation, Funding acquisition, Formal analysis, Data curation, Conceptualization. **Mohd Mudaseer:** Writing – review & editing, Writing – original draft, Visualization, Validation, Supervision, Software, Resources, Project administration, Methodology, Investigation, Funding acquisition, Formal analysis, Data curation, Conceptualization.

## Declaration of competing interest

The authors declare that they have no known competing financial interests or personal relationships that could have appeared to influence the work reported in this paper.
